# A multilevel linear mixed model of the association between candidate genes and weight and body mass index using the Framingham longitudinal family data

**DOI:** 10.1186/1753-6561-3-s7-s115

**Published:** 2009-12-15

**Authors:** Jian'an Luan, Berit Kerner, Jing-Hua Zhao, Ruth JF Loos, Stephen J Sharp, Bengt O Muthén, Nicholas J Wareham

**Affiliations:** 1 Medical Research Council Epidemiology Unit, Institute of Metabolic Science, Addenbrooke's Hospital, Hills Road, Cambridge CB2 0QQ, UK; 2 Center for Neurobehavioral Genetics, Department of Psychiatry and Biobehavioral Sciences, Semel Institute for Neuroscience and Human Behavior, University of California, 695 South Charles E Young Drive, Los Angeles, California 90095, USA; 3 Department of Education, 2005 East Moore Hall, University of California at Los Angeles, California 90095, USA

## Abstract

Obesity has become an epidemic in many countries and is one of the major risk conditions for disease including type 2 diabetes, coronary heart disease, stroke, dyslipidemia, and hypertension. Recent genome-wide association studies have identified two genes (*FTO *and near *MC4R*) that were unequivocally associated with body mass index (BMI) and obesity. For the Genetic Analysis Workshop 16, data from the Framingham Heart Study were made available, including longitudinal anthropometric and metabolic traits for 7130 Caucasian individuals over three generations, each with follow-up data at up to four time points. We explored the associations between four single-nucleotide polymorphisms (SNPs) on *FTO *(rs1121980, rs9939609) or near *MC4R *(rs17782313, rs17700633) with weight and BMI under an additive model. We applied multilevel linear mixed model for continuous outcomes, using the Affymetrix 500k genome-wide genotype data for the four SNPs. The results of the multilevel modeling in the entire sample indicated that the minor alleles of the four SNPs were associated with higher weight and higher BMI. The most significant associations were between rs9939609 and weight (*p *= 4.7 × 10^-6^) and BMI (*p *= 8.9 × 10^-8^). The results also showed that, for SNPs at *FTO*, the homozygotes for the minor allele had the most pronounced increase in weight and BMI, while the common allele homozygotes gained less weight and BMI during the follow-up period. Linkage disequilibrium (LD) between the two *FTO *SNPs was strong (*D*' = 0.997, *r*^2 ^= 0.875) but their haplotype was not significantly associated with either weight or BMI. The two SNPs near *MC4R *were in weak LD (*D*' = 0.487, *r*^2 ^= 0.166).

## Background

As a major contributor to chronic disease including type 2 diabetes, coronary heart disease, stroke, dyslipidemia, and hypertension, obesity has reached epidemic levels in both developed countries and developing countries. It is a multi-factorial disorder that is attributable to multiple genetic and environmental factors. Recent large-scale genome-wide association studies based on cross-sectional population data identified two genes (*FTO *and near *MC4R*) to be unequivocally associated with measures of obesity, including weight and body mass index (BMI) [1-4]. Common variants in the *FTO *gene and near the *MC4R *gene are associated with modest, yet consistent effects on BMI (0.2-0.4 kg/m^2 ^per allele) that translate into odds ratio of 1.1-1.3 for obesity. There is no previous report about this association in longitudinal family data for the two genes.

Here we will analyze the longitudinal family data of the Framingham Heart Study [5] provided for Genetic Analysis Workshop 16, by evaluating associations between weight, BMI, and single-nucleotide polymorphisms (SNPs) on *FTO *(rs1121980, rs9939609) or near *MC4R *(rs17782313, rs17700633), which were recently identified to be consistently associated with obesity-related measures through cross-sectional genome-wide association studies [1,4].

## Methods

The data were provided for 7130 Caucasian individuals across three generations with a maximum of four follow-up exams. Of these, 6,848 had genotype data for at least one of the four SNPs. We focused on weight (kg) and BMI (kg/m^2^) and applied the following criteria for exclusion of individuals: missing data on weight, height, age, or sex (*n *= 100), no family ID (*n *= 227), only measures at <18 years of age (*n *= 1). Measures taken at <18 years of age were excluded from the analysis. The remaining 6520 individuals were part of 962 families, of which 2073 individuals from 697 families had complete data for all four time points. We assumed additive genetic effects (2 = homozygous for the minor allele, 1 = heterozygous for the minor allele, 0 = homozygous for the major allele), with the minor allele being the effect allele for weight or BMI, so that the regression coefficient can be interpreted as the change of weight (kg) or BMI (kg/m^2^) for each additional minor allele.

We used the following three-level model:

where *y*_*kij *_is the weight or BMI at visit *k *for individual *i *within family *j*; *α *a constant; *β*_1_, *β*_2_, and *β*_3 _the effects of SNP, sex, and age, respectively; and *ε*_*kij*_, *γ*_*ij*_, *τ*_*j *_representing individual, family, and between-family error terms. The model was available as *xtmixed *Stata/SE 10.0 for Linux, and was fitted using maximum likelihood with a significance level of *α *= 0.01. Because no gene-sex interaction was observed, a pooled analysis of males and females was done and adjusted for age and sex. To replicate findings in the literature [1,4], we first performed analyses to explore the associations between SNPs and weight or BMI, and then introduced an additional SNP-age interaction term in the model to detect whether age modifies the associations using the full data. The model was then applied using the 2073 individuals with complete data on the four visits to illustrate the change in weight or BMI over time.

We further investigated linkage disequilibrium (LD), as measured by *D*' and *r*^2^, between the SNPs in each of the two genes using the full data. LD measures, haplotype frequencies, and haplotype assignment were calculated, estimated, or reconstructed using SAS/genetics (SAS 9.1.3, SAS Institute Inc., Cary NC). The posterior probabilities of haplotype assignment were 1, 0.999, 0.882, 0.118, and 0.001, respectively, given from SAS procedure. We dropped haplotypes with posterior probability of 0.118 or less, so that each individual has unambiguous assignment of haplotypes. Haplotype analysis was performed in Stata using *xtmixed *procedure while each observation was duplicated as an extra level in *xtmixed*. Haplotypes with haplotype frequency greater than 1% were used for haplotype analysis, adjusted for age and sex.

## Results

Table 1 displays the characteristics of the study participants stratified by generation and sex in each follow-up visit. Overall, 2973 (45.6%) of the participants were men, and 305 (4.7%), 2411 (37.0%), and 3804 (58.3%) of the participants were from the Original, the Offspring and the Generation 3 Cohorts, respectively. The Generation 3 Cohort had measures for only one visit.

**Table 1:** Descriptive characteristics of study participants stratified by sex, generation, and follow-up visit

	*n*		Age (yr) [Mean (SD)]		Weight (kg) [Mean (SD)]		BMI (kg/m^2^) [Mean (SD)]	
				
Cohort	Male	Female	Male	Female	Male	Female	Male	Female
Original (*n *= 305)								
Visit 1	93	211	34.6 (3.1)	34.9 (4.0)	75.2 (10.0)	59.3 (9.1)	25.1 (2.8)	23.2 (3.4)
Visit 2	92	203	40.6 (3.1)	40.9 (4.0)	78.0 (10.2)	61.4 (9.1)	26.0 (2.9)	23.9 (3.4)
Visit 3	90	205	46.6 (3.1)	46.9 (4.1)	78.6 (10.1)	62.6 (9.9)	26.1 (2.7)	24.4 (3.7)
Visit 4	76	184	54.4 (3.1)	54.7 (3.8)	79.3 (9.9)	64.0 (10.2)	27.0 (2.8)	25.0 (3.5)
								
Offspring (*n *= 2411)								
Visit 1	1053	1273	34.4 (8.6)	34.3 (8.8)	81.3 (11.6)	61.8 (11.3)	26.5 (3.4)	23.8 (4.2)
Visit 2	991	1154	46.0 (9.3)	46.4 (9.3)	84.0 (12.5)	65.9 (13.6)	26.9 (3.7)	24.9 (5.0)
Visit 3	1041	1240	53.1 (9.2)	53.3 (9.2)	86.6 (13.4)	69.6 (14.5)	28.0 (4.0)	26.5 (5.4)
Visit 4	1067	1268	60.0 (9.1)	60.3 (9.1)	88.3 (14.7)	71.8 (15.8)	28.6 (4.4)	27.5 (5.9)
								
Generation 3 (*n *= 3804)								
Visit 1	1780	2024	40.3 (8.9)	40.0 (8.8)	88.3 (15.9)	69.9 (16.6)	27.8 (4.7)	25.8 (6.0)

The SNPs were common and in Hardy-Weinberg equilibrium (*p *> 0.28). SNP rs9939609 showed stronger association with BMI (*p *= 8.9 × 10^-8^, Table 2) than rs17700633 (*p *= 0.034). With additive model, the regression coefficient can be interpreted as the change of weight (kg) or BMI (kg/m^2^) for each additional minor allele. It is expected that the weight and BMI will be from 1.26 kg and 0.49 kg/m^2 ^higher for rs9939609 to 0.85 kg and 0.21 kg/m^2 ^higher for rs17700633 if an individual carries one more copy of the minor allele. The results replicate the recent findings in the literature [1,4].

**Table 2:** Association between the SNPs in FTO and near MC4R with weight (kg) and BMI (kg/m^2^)

Gene	SNP	Minor allele	MAF	HWE *p*-value	Weight (kg)			BMI (kg/m^2^)		
						
					Beta	SE	*p*-value	Beta	SE	*p*-value
*FTO*	rs1121980	A	0.44	0.87	1.14	0.27	2.7 × 10^-5^	0.46	0.09	2.6 × 10^-7^
	rs9939609	A	0.40	0.55	1.26	0.27	4.7 × 10^-6^	0.49	0.09	8.9 × 10^-8^
*MC4R*	rs17782313	C	0.22	0.32	1.17	0.32	2.9 × 10^-4^	0.30	0.11	0.004
	rs17700633	A	0.28	0.29	0.85	0.30	0.005	0.21	0.10	0.034

Association analysis was adjusted for age and sex.

**Figure 1:**
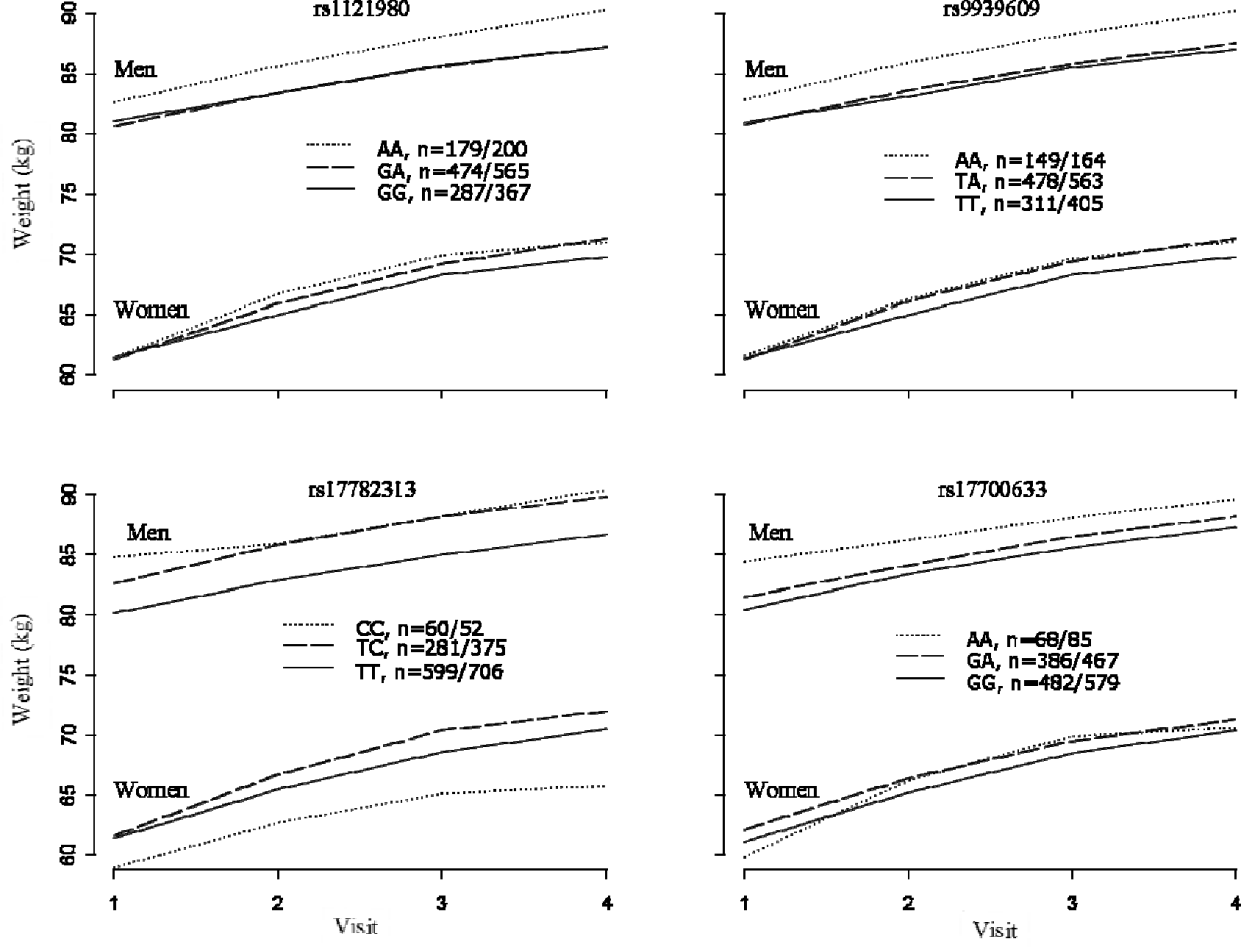
**Follow-up weight of 2073 individuals who had completed data on four time points, adjusted for age**. Genotype frequencies were listed in legends (male/female).

**Figure 2:**
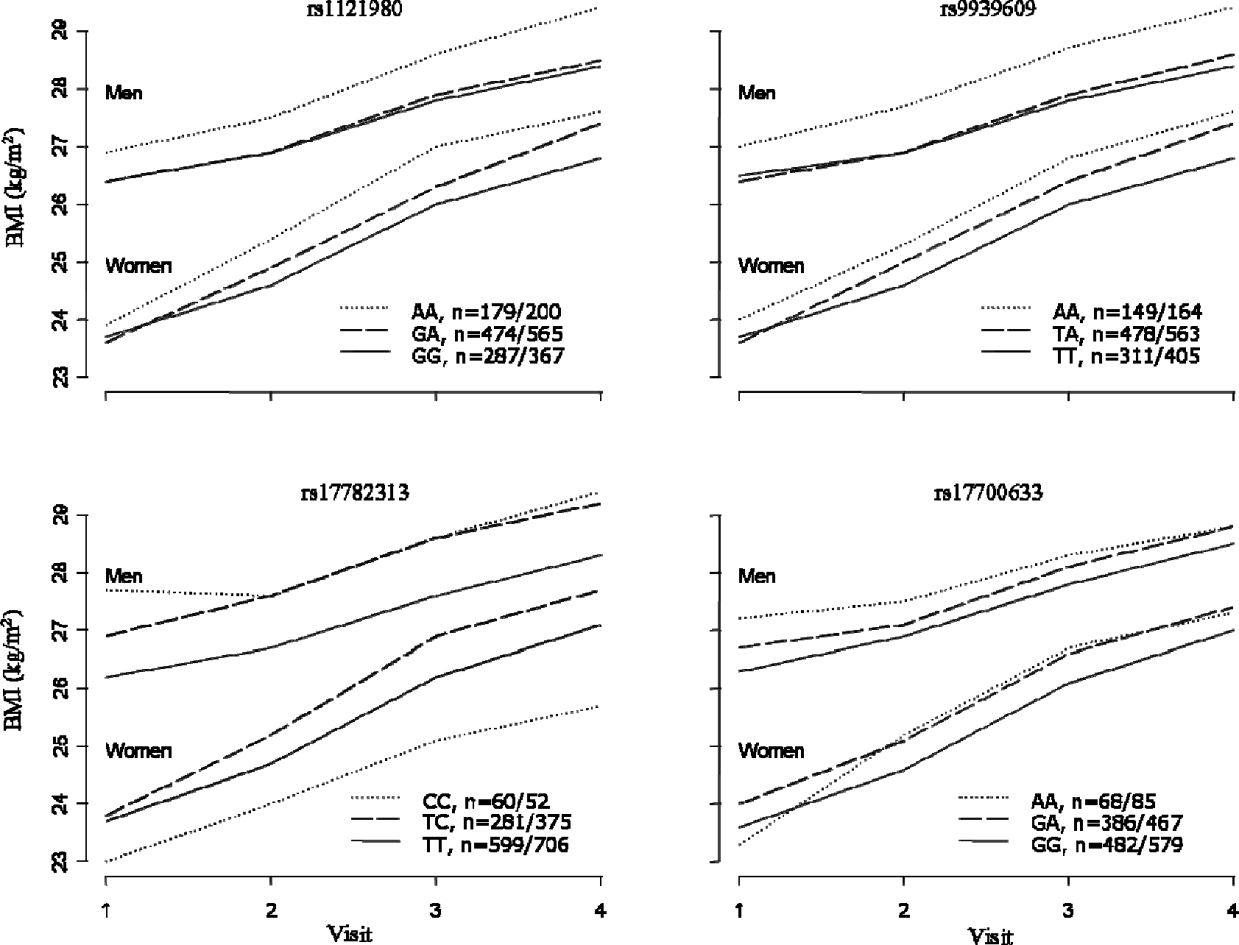
**Follow-up BMI of 2073 individuals who had completed data on four time points, adjusted for age**. Genotype frequencies were listed in legends (male/female).

The SNP-age interactions were significant for *FTO *with both weight and BMI, suggesting that their changes were dependent on the individual's genotype (*p *= 0.0002, 0.0010 for weight, *p *= 0.0001, 0.0010 for BMI, for rs1121980 and rs9939609, respectively). Our results showed that the slopes of weight or BMI with age were significantly different in the three genotypes groups, with homozygotes for the minor allele having a more pronounced increase in weight and BMI over time compared with others genotypes. We found no significant interaction with age for the two SNPs near *MC4R *(*p *> 0.4). The age-adjusted means at each visit by genotype are presented in Figure 1 and Figure 2 for weight and BMI, respectively. Considering a substantial number of individuals had data from only a few visits, especially the Generation 3 Cohort, only individuals with complete data on four time points were used for the illustration in Figure 1 and Figure 2 (*n *= 2073).

The LD measures *D*' and *r*^2 ^were 0.997 and 0.875 between the two *FTO *SNPs, and 0.487 and 0.166 between the two near *MC4R *SNPs, respectively. We performed the haplotype analysis for the two *FTO *SNPs, which showed strong evidence of LD. The estimated haplotype frequencies for rs1121980 and rs9939609 were: 0.403 (A-A), 0.032 (A-T), 0.001 (G-A), and 0.564 (G-T). The G-A haplotype was excluded from further analysis because its haplotype frequency was less than 1%. There was no significant interaction between haplotype and age, *p *= 0.69 and *p *= 0.44 respectively, for BMI and weight. The results of haplotype analyses showed no significant association for *FTO *haplotype with BMI (*p *= 0.817) or with weight (*p *= 0.351).

## Discussion

We have analyzed the Framingham longitudinal family data by considering two SNPs on the *FTO *gene and two near the *MC4R *gene. As a first step, we analyzed the entire sample and considered it as a homogeneous group. With the exception of only one SNP (rs17700633) on BMI, our linear mixed models confirmed that the minor alleles of the SNPs rs1121980, rs9939609, and rs17782313 were significantly associated with higher weight and BMI. The SNPs on the *FTO *gene were in stronger association with both weight and BMI than SNPs near *MC4R*.

From investigations of change of weight and BMI over time, we found that for both SNPs on the *FTO *gene, the homozygotes for the minor allele had the most pronounced increase in weight and BMI. Although the SNPs near the *MC4R *gene showed cross-sectional association with weight and BMI across visits, we found no evidence that the difference between genotypes increased over time. These observations may have important implications for treatment and prevention of obesity at different stages of life. Our results suggest that genetic susceptibility to weight gain induced by the *FTO *SNPs rs1121980 and rs9939609 increases with age. The latter observation can only be made using studies with a longitudinal design.

The lack of association of *FTO *haplotypes with weight and BMI is somewhat counterintuitive. However, similar results were obtained when the haplotype analysis was repeated using the FAMHAP software [6]. When both SNPs were included in a single model, neither SNP was significant (data not shown).

Our work has both methodological and practical merits, with application to whole genome-wide data involving quantitative and longitudinal quantitative trait analyses.

## List of abbreviations used

BMI: Body mass index; LD: Linkage disequilibrium; SNP: Single-nucleotide polymorphism

## Competing interests

The authors declare that they have no competing interests.

## Authors' contributions

JAL designed the study, performed the analysis, and drafted the manuscript. BK contributed to the study design, statistical experiment, and provided comments on the manuscript. J-HZ proposed the study, arranged to obtain the GAW16 Framingham Heart Study data, and participated in the analysis, discussion, and final revision of the manuscript. SJS and RJFL contributed to the study design, statistical models, and manuscript drafting. NJW and BOM supervised the study design and the experimental work. All authors read and approved the final manuscript.
